# Supplementary figures and images for: Live and heat-killed Leuconostoc mesenteroides counteract the gastrointestinal dysfunction in chronic kidney disease mice through intestinal environment modulation
Source: PLoS One. 2025 Feb 24;20(2):e0318827. doi: 10.1371/journal.pone.0318827 (PMC12005673; doi:10.1371/journal.pone.0318827)

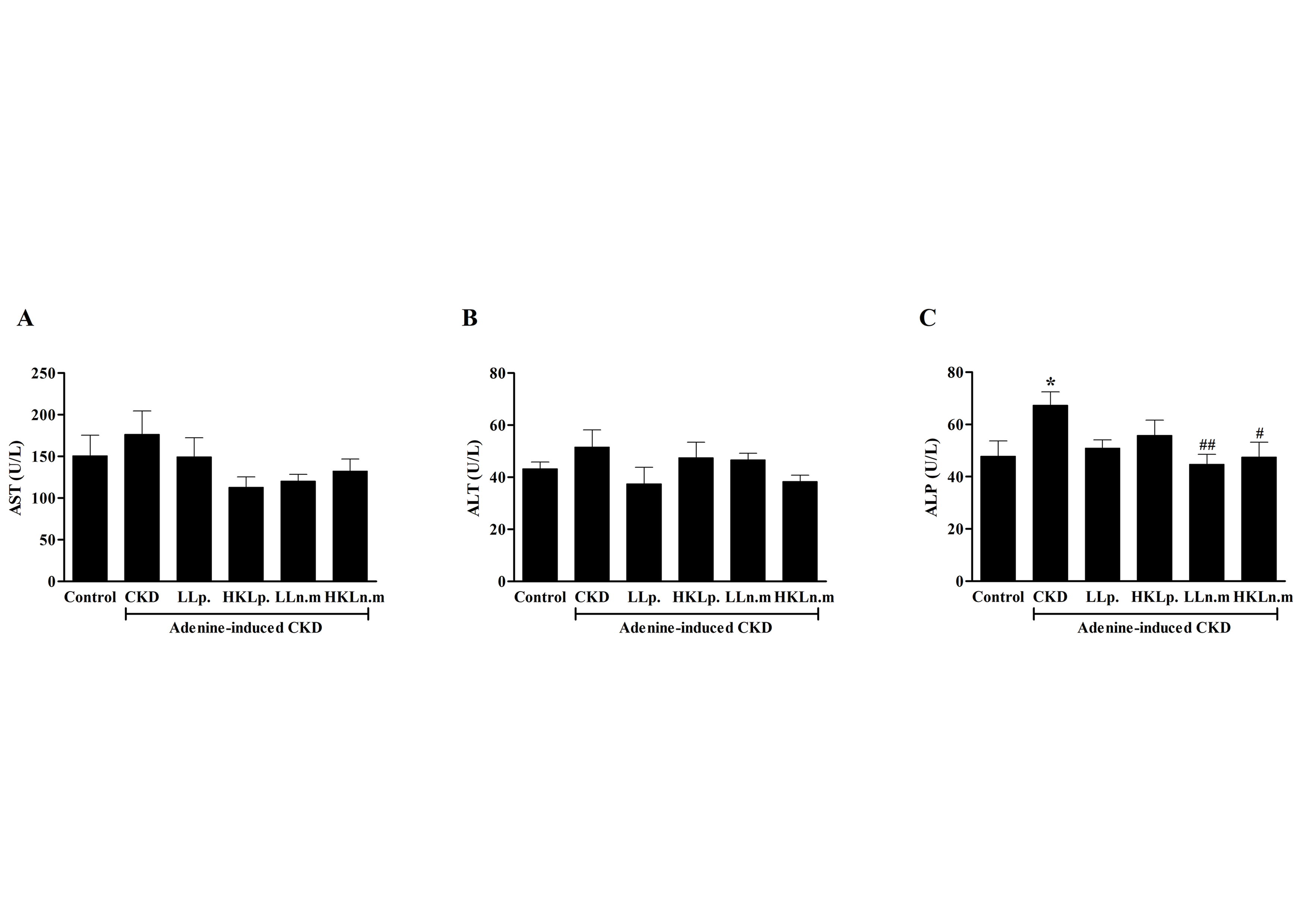

Supplement: S1 Fig — The liver function was investigated by enzyme levels after the treatment period. A, B, and C: The AST, ALT, and ALP levels, respectively. CKD; chronic kidney disease, LLp; live Lactiplantibacillus plantarum, HKLp; Heat-killed Lactiplantibacillus plantarum, LLn.m; Live Leuconostoc mesenteroides, and HKLn.m; Leuconostoc mesenteroides. Data were shown as mean ± SEM (n = 7–10). #p < 0.05 when compared to the control group; *p < 0.05 and **p < 0.01 when compared to the CKD group (one-way ANOVA repeated by Bonferroni test). (TIF) [file pone.0318827.s001.tif]

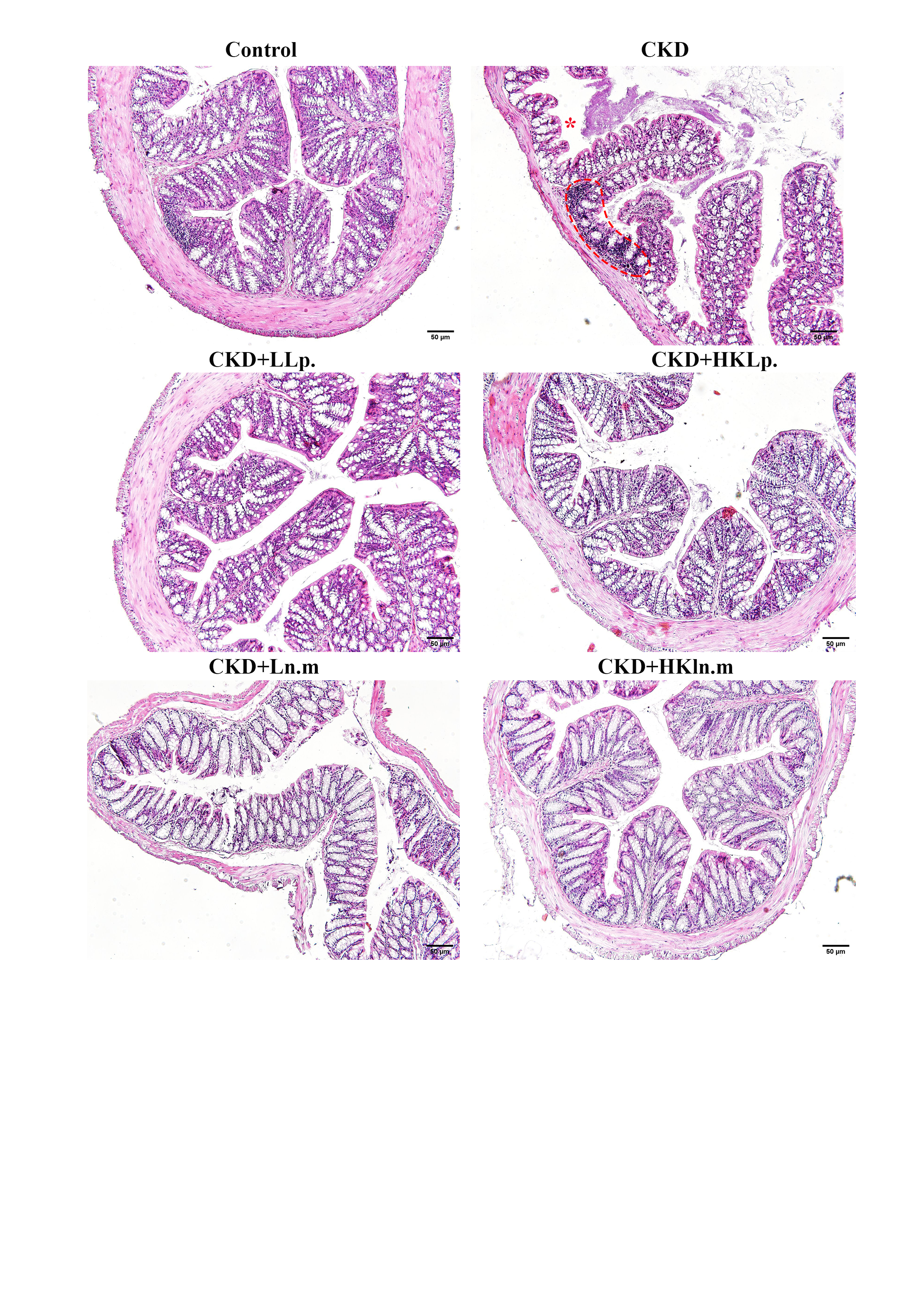

Supplement: S2 Fig — The H & E staining of small intestinal tissue was stained with H & E to investigate the histological morphology of the large intestine in CKD mice. The structure of the colon was the same way with the small intestine, the tissue staining showed mucosal damage and inflammation in the CKD group. While in CKD treated with probiotic groups ameliorated this effect of CKD on the colon. CKD; chronic kidney disease, LLp; live Lactiplantibacillus plantarum, HKLp; Heat-killed Lactiplantibacillus plantarum, LLn.m; Live Leuconostoc mesenteroides, and HKLn.m; Leuconostoc mesenteroides. (TIF) [file pone.0318827.s002.tif]
