# Supplementary material for: Live and heat-killed Leuconostoc mesenteroides counteract the gastrointestinal dysfunction in chronic kidney disease mice through intestinal environment modulation
Source: PLoS One. 2025 Feb 24;20(2):e0318827. doi: 10.1371/journal.pone.0318827 (PMC12005673; doi:10.1371/journal.pone.0318827)
Supplement: S1 Table — (TIF) [file pone.0318827.s003.pdf]

S1 Table: Antibiotic susceptibility of the isolated lactic acid bacteria

Antibiotics	Clear zone diameter (mm)																							
	L. plantarum		*L. casei*		P. 6.1		P. 6.4		P. 6.6		P. 7.1		P. 7.2		P. 7.4		P. 7.5		P. 9.1		P. 10.1		P. 11.4	
**Amikacin**	10	R	13	R	16	I	20	I	16	I	11	R	10	R	13	R	15	R	14	R	15	R	14	R
**Ampicillin**	41	S	34	S	28	S	29	S	29	S	36	S	28	S	23	S	31	S	25	S	29	S	28	S
**Aztreonam**	0	R	0	R	0	R	0	R	0	R	7.2	R	0	R	0	R	0	R	0	R	0	R	0	R
**Cefoxitin**	33	S	19	I	16	I	16	I	15	R	14	R	16	I	9.7	R	17	I	14	R	17	I	15	R
**Ceftriaxone**	0	R	29	S	13	R	13	R	11	R	19	I	21	S	15	R	28	S	21	S	21	S	19	I
**Chloramphenicol**	40	S	36	S	36	S	36	S	35	S	28	S	34	S	30	S	31	S	26	S	31	S	30	S
**Ciprofloxacin**	10	R	13	R	17	I	17	I	17	I	14	R	15	R	14	R	19	I	17	I	18	I	19	I
**Clindamycin**	15	R	31	S	32	S	32	S	33	S	26	S	33	S	33	S	32	S	28	S	28	S	28	S
**Erythromycin**	36	S	44	S	34	S	32	S	35	S	25	S	32	S	30	S	33	S	25	S	29	S	28	S

Antibiotics	Clear zone diameter (mm)																							
	L. plantarum		*L. casei*		P. 6.1		P. 6.4		P. 6.6		P. 7.1		P. 7.2		P. 7.4		P. 7.5		P. 9.1		P. 10.1		P. 11.4	
**Gentamycin (30)**	15	R	15	R	20	I	20	I	21	S	13	R	13	R	11	R	15	R	14	R	15	R	15	R
**Gentamycin (10)**	12	R	16	I	16	I	16	I	15	R	11	R	15	R	0	R	14	R	13	R	13	R	13	R
**Kanamycin**	0	R	0	R	12	R	12	R	14	R	0	R	11	R	0	R	13	R	13	R	11	R	12	R
**Streptomycin**	0	R	14	R	12	R	12	R	11	R	0	R	14	R	0	R	0	R	12	R	11	R	14	R
**Sulbactam**	36	S	33	S	31	S	31	S	29	S	26	S	25	S	21	S	26	S	25	S	27	S	23	S
**Suphamethoxazole**	19	I	0	R	0	R	0	R	0	R	0	R	0	R	0	R	0	R	0	R	0	R	0	R
**Tetracycline**	34	S	46	S	38	S	36	S	41	S	30	S	33	S	30	S	32	S	28	S	31	S	30	S
**Vancomycin**	0	R	0	R	0	R	0	R	0	R	7.2	R	9.3	R	0	R	0	R	0	R	0	R	0	R

Inhibition zone diameters were measured inclusive of the diameter of the discs. Results were expressed as sensitive, S (≥ 21 mm); intermediate, I (16-20 mm) and resistant, R (≤ 15 mm), respectively according to that described by Liasi et al., 2009. *L. plantarum*; *Lactiplantibacillus plantarum*, *L. casei*; *Lactobacillus casei*.
