# Supplementary material for: Live and heat-killed Leuconostoc mesenteroides counteract the gastrointestinal dysfunction in chronic kidney disease mice through intestinal environment modulation
Source: PLoS One. 2025 Feb 24;20(2):e0318827. doi: 10.1371/journal.pone.0318827 (PMC12005673; doi:10.1371/journal.pone.0318827)
Supplement: S2 Table — (PDF) [file pone.0318827.s004.pdf]

S2 Table: Effects of heat-inactivated *Leuconostoc mesenteroides* on hematological parameters

Groups/ Parameters	Control	CKD	CKD + LLp.	CKD + HKLp.	CKD + Ln.m	CKD + HKLn.m
**WBC ($x\ 10^{3}$ / uL)**	3.15 ± 1.03	2.71 ± 0.50	2.48 ± 0.40	3.60 ± 1.08	2.44 ± 0.33	3.91 ± 1.42
**Lymphocyte ($x\ 10^{3}$/ uL)**	0.93 ± 0.23	0.63 ± 0.15	0.60 ± 0.11	2.11 ± 0.80	0.78 ± 0.30	1.84 ± 1.07
**Monocyte ($x\ 10^{3}$/ uL)**	0.13 ± 0.06	0.08 ± 0.03	0.21 ± 0.12	0.10 ± 0.03	0.11 ± 0.02	0.20 ± 0.07
**Granulocyte ($x\ 10^{3}$/ uL)**	2.10 ± 0.99	2.00 ± 0.54	1.80 ± 0.40	1.40 ± 0.36	1.56 ± 0.25	1.90 ± 0.55
**RBC ($x\ 10^{6}$/uL)**	8.50 ± 0.50	7.38 ± 0.50	7.70 ± 0.50	6.65 ± 0.75	7.50 ± 0.31	7.20 ± 0.43
**HGB (g/dL)**	14.40 ± 0.46	13.00 ± 0.90	12.97 ± 0.93	12.44 ± 0.98	13.50 ± 0.60	12.10 ± 0.64
**HCT (%)**	42.97 ± 1.98	37.01 ± 2.23	38.30 ± 2.72	33.04 ± 4.00	37.00 ± 1.50	35.52 ± 2.00
**MCV (fL)**	50.97 ± 1.25	50.42 ± 1.20	46.16 ± 3.00	49.60 ± 0.65	49.61 ± 0.43	49.63 ± 1.26
**MCH (pg)**	17.1 ± 0.69	17.60 ± 1.02	16.90 ± 0.97	19.37 ± 1.50	18.10 ± 0.70	17.04 ± 1.00
**MCHC (g/dL)**	33.65 ± 1.17	39.83 ± 4.90	34.3 ± 2.21	39.27 ± 3.01	36.55 ± 1.25	34.66 ± 2.04
**PLT ($x\ 10^{3}$/ uL)**	1157.96 ± 105.42	1443 ± 151.60	1318.90 ± 217.00	1544.50 ± 413.50	1656.70 ± 83.70	1317.40 ± 237.45

**MPV (fL)**	4.84 ± 0.10	4.70 ± 0.06	4.71 ± 0.08	4.70 ± 0.15	4.60 ± 0.05	5.30 ± 0.43

CKD: chronic kidney disease, LLp.: live *Lactiplantibacillus plantarum*, HKLp.: Heat-killed *Lactiplantibacillus plantarum*, LLn.m: Live *Leuconostoc mesenteroides*, and HKLn.m: *Leuconostoc mesenteroides*. WBC; white blood cell, RBC; red blood cell, HBG; hemoglobin, HCT; hematocrit, MCV; mean corpuscular volume, MCH; mean corpuscular hemoglobin, MCHC; mean corpuscular hemoglobin concentration, PLT; platelets, and MPV; mean platelet volume. Data was shown as mean ± SEM (n= 6–10). (one-way ANOVA repeated by Bonferroni test).
