# Supplementary material for: Live and heat-killed Leuconostoc mesenteroides counteract the gastrointestinal dysfunction in chronic kidney disease mice through intestinal environment modulation
Source: PLoS One. 2025 Feb 24;20(2):e0318827. doi: 10.1371/journal.pone.0318827 (PMC12005673; doi:10.1371/journal.pone.0318827)
Supplement: S3 Table — (PDF) [file pone.0318827.s005.pdf]

S3 Table: Nutritional composition of mice feed

Nutrient	Value
Moisture (Max)	12%
Crude Protein (Min)	24%
Fat (Min)	4.5%
Fiber (Max)	5%
Metabolizable Energy	3,040 Kcal/kg
Calcium	1.0%
Phosphorus (Available)	0.9%
Sodium	0.20%
Potassium	1.17%
Magnesium	0.23%
Manganese (p.p.m.)	171
Copper (p.p.m.)	22
Zinc (p.p.m.)	100
Iron (p.p.m.)	180
Cobalt (p.p.m.)	1.82
Potassium Iodide (p.p.m.)	-
Selenium (p.p.m.)	0.1

Vitamins

Vitamin	Unit	Value
Vitamin A	i.u. / kg.	20,000
Vitamin D3	i.u. / kg.	4,000
Vitamin E	mg / kg.	100
Vitamin K	mg / kg.	5
Vitamin B1 (Thiamine)	mg / kg.	20
Vitamin B2 (Riboflavin)	mg / kg.	20
Vitamin B6	mg / kg.	20

Vitamin B12	mg / kg.	0.036
Niacin	mg / kg.	100
Folic Acid	mg / kg.	6
Biotin	mg / kg.	0.4
Pantothenic Acid	mg / kg.	60
Choline Chloride	mg / kg.	1,500
